# Monte Carlo simulations of microtubule arrays: The critical roles of rescue transitions, the cell boundary, and tubulin concentration in shaping microtubule distributions

**DOI:** 10.1371/journal.pone.0197538

**Published:** 2018-05-21

**Authors:** Lynne Cassimeris, Jessica C. Leung, David J. Odde

**Affiliations:** 1 Dept. of Biological Sciences, Lehigh University, Bethlehem, Pennsylvania, United States of America; 2 Dept. of Biomedical Engineering, University of Minnesota, Minneapolis, Minnesota, United States of America; University of Illinois at Chicago, UNITED STATES

## Abstract

Microtubules are dynamic polymers required for a number of processes, including chromosome movement in mitosis. While regulators of microtubule dynamics have been well characterized, we lack a convenient way to predict how the measured dynamic parameters shape the entire microtubule system within a cell, or how the system responds when specific parameters change in response to internal or external signals. Here we describe a Monte Carlo model to simulate an array of dynamic microtubules from parameters including the cell radius, total tubulin concentration, microtubule nucleation rate from the centrosome, and plus end dynamic instability. The algorithm also allows dynamic instability or position of the cell edge to vary during the simulation. Outputs from simulations include free tubulin concentration, average microtubule lengths, length distributions, and individual length changes over time. Using this platform and reported parameters measured in interphase LLCPK1 epithelial cells, we predict that sequestering ~ 15–20% of total tubulin results in fewer microtubules, but promotes dynamic instability of those remaining. Simulations also predict that lowering nucleation rate will increase the stability and average length of the remaining microtubules. Allowing the position of the cell’s edge to vary over time changed the average length but not the number of microtubules and generated length distributions consistent with experimental measurements. Simulating the switch from interphase to prophase demonstrated that decreased rescue frequency at prophase is the critical factor needed to rapidly clear the cell of interphase microtubules prior to mitotic spindle assembly. Finally, consistent with several previous simulations, our results demonstrate that microtubule nucleation and dynamic instability in a confined space determines the partitioning of tubulin between monomer and polymer pools. The model and simulations will be useful for predicting changes to the entire microtubule array after modification to one or more parameters, including predicting the effects of tubulin-targeted chemotherapies.

## Introduction

The microtubule (MT) cytoskeleton is a major driver of cell polarization and intracellular organization. The MT cytoskeleton is formed from hundreds of linear polymers, each assembled from tubulin protein subunits. This MT polymer system is able to reorganize itself, responding to cues such as the position of the plasma membrane or cell cycle timing, to change the lengths and turnover of individual MT polymers. MTs serve as the tracks for the motor proteins that power directed movement of cargo, such as membrane vesicles to the plasma membrane for secretion. MTs also form the mitotic spindle during mitosis; this structure is responsible for accurately segregating the replicated genome at each cell division. The MT cytoskeleton has been a highly successful target for chemotherapies used to treat multiple cancers, while mutations in some tubulin subunits have been linked to Amyotrophic Lateral Sclerosis (ALS) or neurological development disorders [1,2]. Here we describe an algorithm to simulate the array of dynamic MTs and to follow reorganization of the array as conditions change.

Individual MTs rapidly exchange subunits with a soluble pool of alpha/beta tubulins, allowing individual MTs to explore space within the cytoplasm (e.g. to connect to kinetochores of chromosomes during mitosis) or allowing the entire MT array to reorganize rapidly in response to external or internal cues. MT polymers turn over by dynamic instability, which is most simply defined as phases of growth (net tubulin addition to polymer ends) and shortening (net tubulin loss from polymer ends), with abrupt, infrequent transitions between these phases termed catastrophe (growth to shortening) and rescue (shortening to growth) [3–5]. Additional states include short-term pauses, where MTs show little net change in length on the order of seconds, to stable, non-dynamic MTs that maintain a constant length for hours [6,7]. Since the 1980's, the parameters of MT plus end dynamic instability have been measured at the cell periphery in a wide array of cell types [8–11]. More recently, EB1-GFP has been used as a marker of MT plus end polymerization, allowing measurement of both nucleation from the centrosome and growth throughout the cytoplasm. Computer-based tracking algorithms then infer the other parameters of dynamic instability from the disappearance or re-appearance of EB1-GFP "comets" at MT tips [10–14]. From these analyses, the functions of numerous proteins to regulate one or more parameters of dynamic instability have been described, as well as changes to dynamic instability in different cell cycle stages (interphase vs mitosis) [9], different regions within the cell (e.g. leading and trailing edges of motile cells [15]), or in response to MT, or tubulin-targeted, drugs [16–18].

While we have a good understanding of how specific proteins modulate dynamic instability, with a growing understanding of molecular mechanisms of protein-protein interactions that are responsible for shaping tubulin polymerization into MTs, we currently do not have a way to take a more distant view and explore how the combined parameters of dynamic instability function together to shape an entire array of MTs, and then look at how changes to dynamic parameter(s) shifts the array’s organization over time and space. Previous models [3,18–21] focused on interphase MTs, and were often not designed to follow an entire MT array as the system shifts to a new steady-state. To explore MT length distributions and numbers, monomer/polymer partitioning and the search area of the dynamic plus ends, we developed a relatively simple Monte Carlo model to simulate hundreds of MTs undergoing dynamic instability within a confined space mimicking cellular dimensions. The simulations are blind to molecular mechanisms underlying the measured dynamics parameters. These simulations allowed us to examine how an array shifts from one distribution to another, such as an interphase array to a prophase array. Simulations predict that rescue frequency and total tubulin concentration are major factors dictating the number of microtubules and the shape of the interphase array. The position of the boundary/cell edge is also critical during interphase (see also, [10,20]), while decreased rescue frequency is the sole factor required to depolymerize the interphase array prior to assembly of the mitotic spindle. Our simulations yield several counter-intuitive predictions of how the MT system responds to changes in nucleation rate or total tubulin concentration (see also [20]), providing the cell with mechanisms to shift the numbers or lengths of microtubules independent of direct binding of regulatory proteins to MT plus ends.

## Results and discussion

### MT organization in interphase LLCPK1 cells

**Fig 1:**
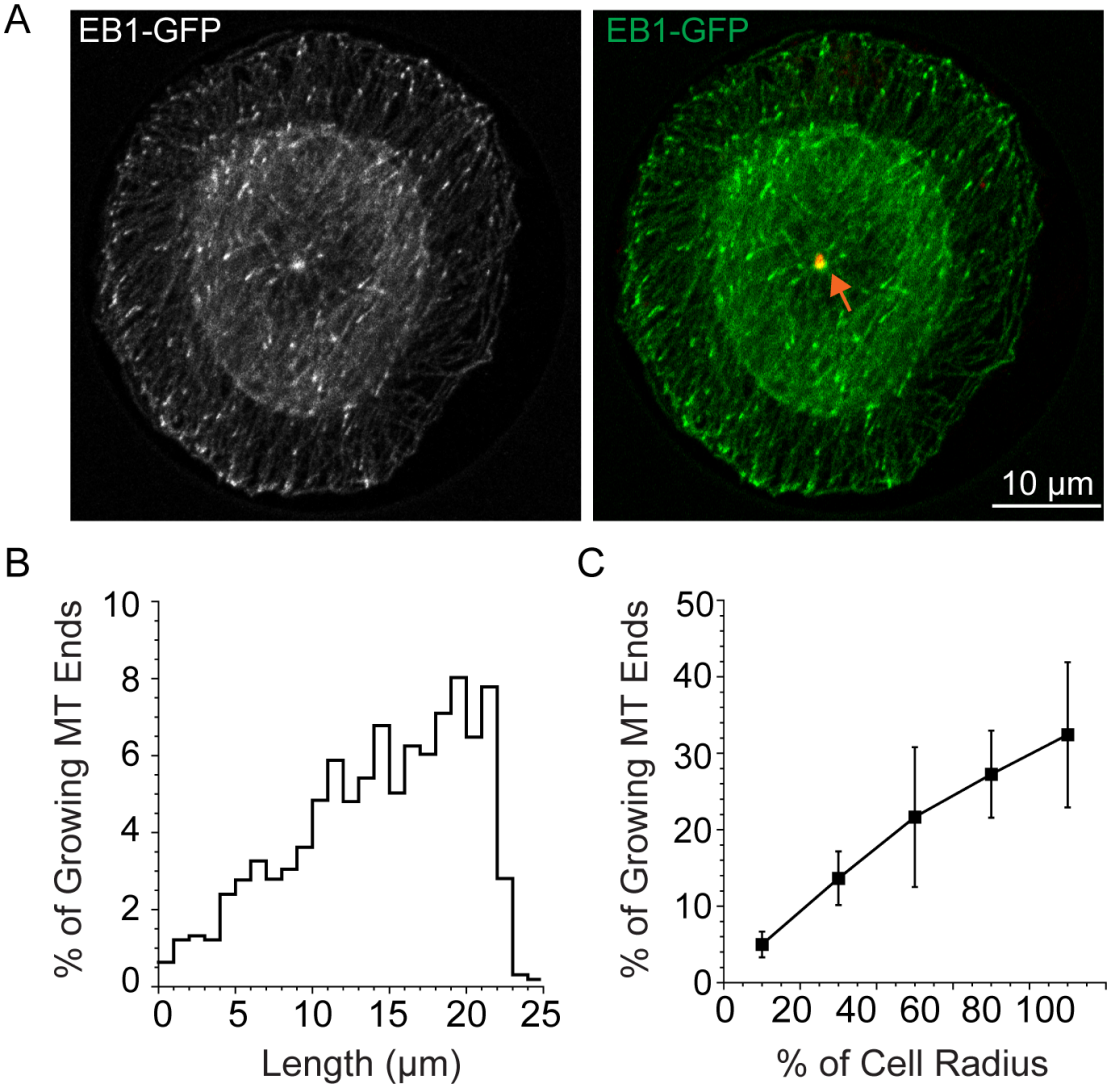
MT length distributions for growing MT ends in LLCPK1 cells. (A) Cells were fixed 4 h after plating on a disc shaped adhesive surface. The position of the centrosome is shown in red and marked by the orange arrow. EB1-GFP marks growing MT plus ends. Lengths were measured assuming straight lines between the center of the centrosome and the distal tip of the EB1-GFP comet (B) Distribution of MT lengths averaged for 4 cells plated on a disc shaped pattern and having centered centrosomes.

As a reference point for comparison to our simulations, we measured lengths of growing MTs, marked by EB1 at their tips, for LLCPK1 cells. We chose to use LLCPK1 cells here and for the parameters used in the simulations below because this epithelial cell line has been used extensively for study of MT plus end dynamics and nucleation at the centrosome [9,16,18,22–24]. Both free tubulin (7 μM) and total tubulin (35 μM) have also been measured recently for this cell type [18,22]. We measured growing MTs from the basal section closest to the coverslip for 4 cells attached to a disk shaped adhesive pattern of 22.6 μm radius (Fig 1). These cells were selected because the centrosome is centered nearly perfectly in each of these cells (Fig 1A). Using the basal section and cells with a centered centrosome allowed us to measure a distribution of MT lengths that is most closely related to the array we simulate below. Fig 1B shows the length distribution of MTs in these cells. The distribution of growing MT ends rises nearly linearly with cell radius, and then decays rapidly to a small percent of MT ends near the cell margin. When plotted as a percentage of cell radius and binned into 5 equal fractions, the lengths fall into a nearly straight line, with longer MTs more abundant than shorter ones. Measuring the lengths of EB1-marked MT tips from the centrosome assumes that MTs are growing in straight lines, which is a reasonable approximation for the typical radial array of interphase MTs. EB1-GFP labels the tips of ~90% of MTs at the cell periphery [25] indicating that the length distributions measured by EB1 localizations provides a convenient marker for the great majority of MTs in the interphase array.

### Monte Carlo simulation of an interphase MT array

To simulate an array of MTs, we developed a model where MTs are nucleated from a defined number of sites located in the center of a cylinder with a radius of 25 μm and a height of 0.5 μm for a volume of approximately 1000 μm^3^ (Table 1). This simple shape mimics the basal region of a typical cell attached to a coverslip where most MTs are located and where MT dynamics are typically measured. General simplifying features of the model are given in Methods. The concentration of total tubulin was typically set to 35 μM [18,23]. For most simulations the number of nucleation sites was set to 500, which also sets the maximum number of MTs. Additional assumptions were: (1) complete MT depolymerization immediately opens up that nucleation site for a new nucleation event, which occurs with a probability dictated by the measured nucleation rate [22]; (2) all MTs remain associated with their nucleation site until they completely depolymerize and that MTs grow in straight lines and do not bend when they encounter the cell margin; (3) MT growth rate is dependent on free tubulin concentration; (4) shortening rate, and catastrophe and rescue frequencies are not dependent on tubulin concentration; (5) MTs grow in straight lines and do not bend when they encounter the cell margin; (6) any MT reaching the cell boundary undergoes a catastrophe.

**Table 1:** Common simulation parameters.

Parameter	Value
Nucleation Sites	500
Nucleation Rate/Site	0.0005 s^-1^
Total Tubulin	35 μM
Cell Radius	25 μm
k_on_	0.0167 μm μM^-1^ s^-1^
k_off_	0 μm/s

Simulations, with each step simulating 1 s of time based, followed the general sequence: (1) available sites are tested for nucleation based on the given probability, (2) if nucleated, microtubules are put in a growing state, with a velocity determined by the free tubulin concentration (note that the first growth step occurred at a defined rate allowing MT growth to initiate but the growth rate was dependent on tubulin concentration for all steps thereafter), (3) growing MTs can remain in a growing state or undergo a catastrophe, thereby switching the MT to a shortening state. MTs that reach the cell boundary also transition to a shortening state. The sequence continues: (4) shortening MTs can continue to shorten or undergo a rescue event and resume growth. Output from the simulations include MT number and lengths, free tubulin, and the length history of a single MT over the course of the simulation.

Common parameters and values used to simulate the interphase MT array. Nucleation rate per site was estimated based on published rates of new EB1-GFP labeled MT tips emerging from the centrosome [22] divided by the 500 potential sites. Total tubulin concentration was estimated as described previously [18,23]. The apparent rate constants, k_on_ and k_off_, were used to calculate growth rate as a function of free tubulin concentration (see Methods). The values listed for each parameter were used unless stated otherwise.

**Fig 2:**
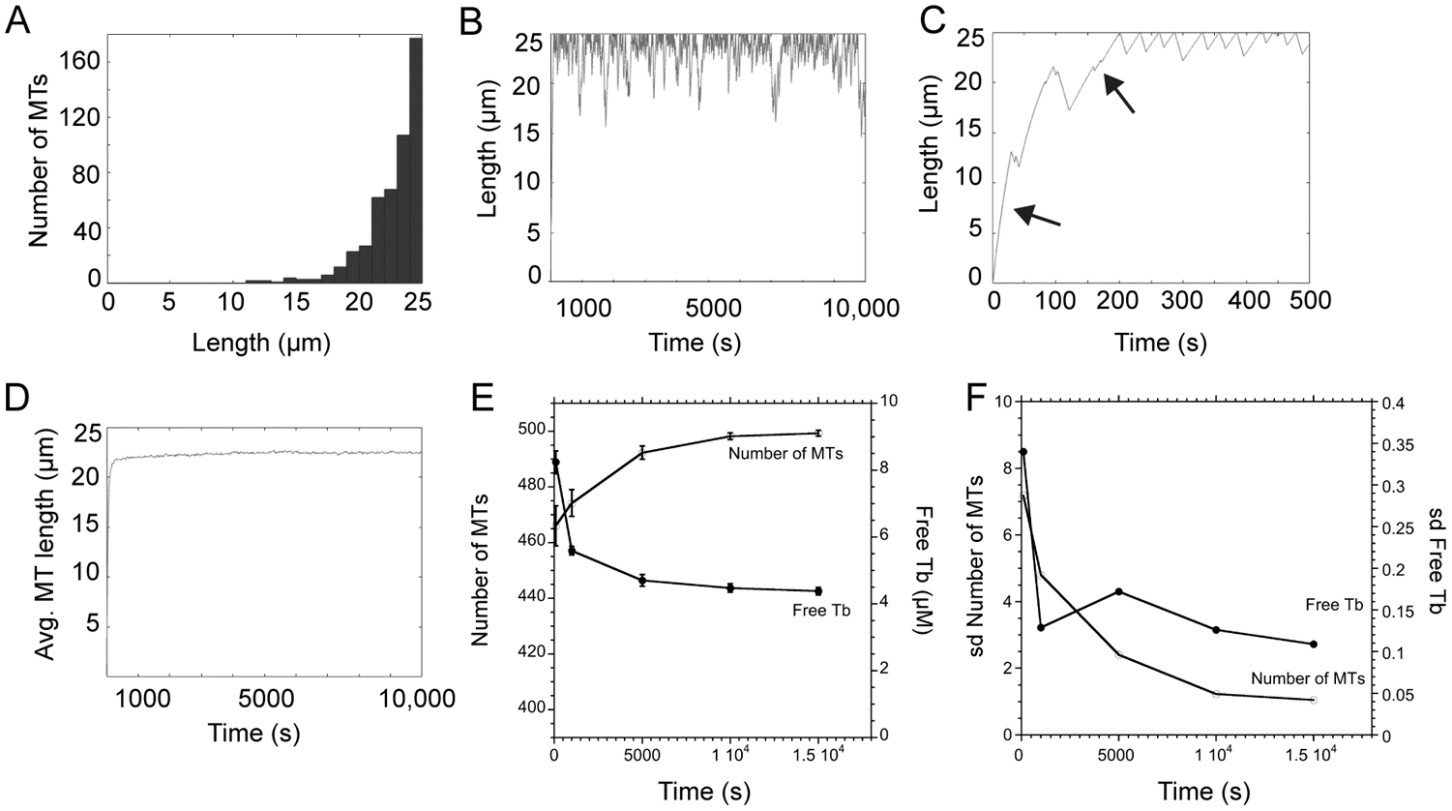
Parameter Set A generates an array of long MTs, confined by the cell boundary. (A) Simulated MT length distribution for a radius of 25 μm. (B) Length of a single simulated MT over the 10,000 s of the simulation run. (C) The first 500 s of a simulation is shown to highlight the slower rate of MT polymerization as tubulin assembles into polymer (compare slopes at arrows). Free tubulin concentration declines rapidly during this time course (see graph in E). (D) Average MT length reaches a stable value of ~22 μm rapidly during simulations. (E,F) The number of MTs and free tubulin concentration, as well as their standard deviations, reach plateaus by ~ 10,000 s.

The first parameter set examined was based on data for interphase LLCPK1 cells, with values for nucleation rate [22] and plus end dynamic instability (Parameter Set A; [9]) as listed in Table 2. Fig 2 shows the output from simulations run for 10,000 s (2.8 h of MT dynamics within a cell). As shown in Fig 2A, the distribution of MT lengths forms a steeply rising exponential, where MT ends are confined to a region near the edge, as defined by the cell radius. The length of a single MT over the course of the simulation is shown in Fig 2B. Consistent with the distribution of all MT ends, the end of this single MT spends most of its time near the cell boundary, rarely shortening more than 5 μm from the edge. Fig 2C shows a simulation run for fewer steps to show the early steps in MT assembly for a single MT. Slowing of growth rate, as tubulins are incorporated into polymer, was evident by changes in the slope over the first few growth phases. Average MT length rapidly reached about 22 μm (Fig 2D). The total number of MTs increased more slowly to nearly 500 MTs by about 5,000 s, with a concomitant decline in free tubulin to 4–5 μM over this same time course (Fig 2E). We used 10,000 s for most simulations reported here because the number of MTs and free tubulin concentration reach approximate steady state values by this time (Fig 2E) and the standard deviations of these values also reach plateaus (Fig 2F). Oosawa [26] previously demonstrated that the standard deviation is slower to relax than the mean, making the plateau in standard deviations the more appropriate measure that simulations were of sufficient duration to generate steady state values.

**Table 2:** Parameter sets for MT plus end dynamic instability.

	Set A	Set B	NEBD
V_g_ (μm s^-1^)	0.192 ± 0.123	0.142 ± 0.097	0.178± 0.153
V_s_ (μm s^-1^)	0.218 ± 0.144	0.188 ± 0.132	0.205 ± 0.087
k_c_ (s^-1^)	0.026 ± 0.024	0.053 ± 0.003	0.075 ± 0.089
k_r_ (s^-1^)	0.175 ± 0.104	0.086 ± 0.005	0.023 ± 0.029

Dynamic instability parameters (V_g_, growth rate; V_s_, shortening rate; k_c_, catastrophe frequency; k_r_, rescue frequency) for Set A [9], Set B [27] and at NEBD/prophase [9] measured in LLCKP1 cells expressing GFP-alpha tubulin.

Overall, parameter set A generated MTs that grew to long lengths and quickly filled all, or nearly all, available nucleation sites. The length distributions derived from Parameter Set A did not match well with the measured distributions (Fig 1). The combination of parameters generates an array of long MTs that are unlikely to ever depolymerize completely, which means that new nucleation sites would not open up at a rate sufficient to generate the experimentally observed rate of EB1-GFP comet emergence from centrosomes. To test this interpretation, we used a 2-state simulation where we built a MT array using parameter set A in state 1 and then set nucleation rate to zero in state 2. Simulations were run for various times in state 2. For these parameters, the MT array assembled in state 1 was stable in state 2, losing only ~ 15 of the original 498 MTs after 173,000 s (48 h) demonstrating that MTs generated in state 1 rarely depolymerize completely (S1 Fig).

**Fig 3:**
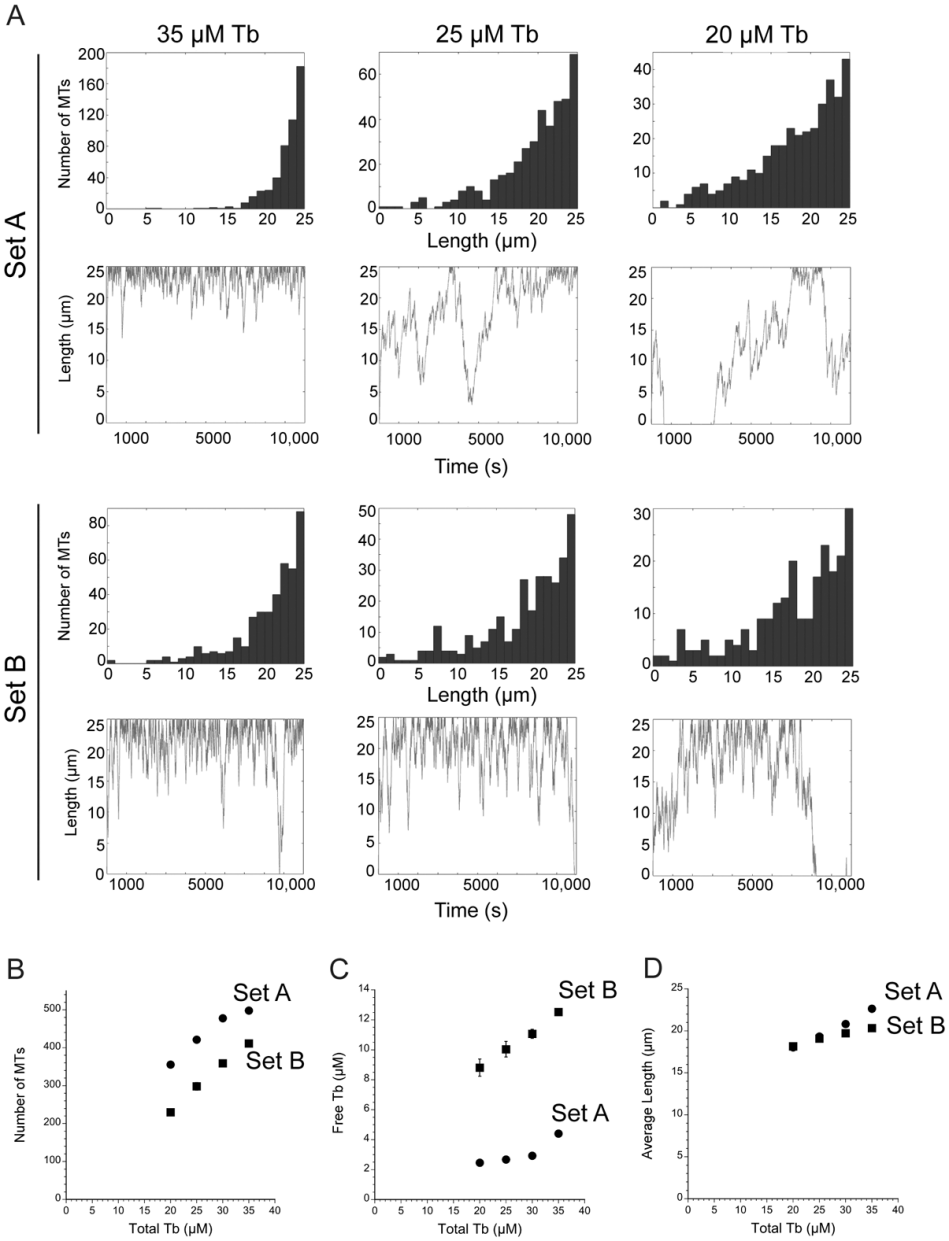
Comparison of outputs from parameter Sets A and B with varying total tubulin concentrations. (A) Simulation outputs as noted for parameter sets A and B and indicated total concentrations of tubulin. (B-D) Average ± sd values for MT number, free tubulin concentration and average MT length as a function of total tubulin concentration. Standard deviations are often smaller than the size of the data point.

Since parameter set A generated MTs that were overly stable, we simulated MT arrays generated by a second published set of parameters from LLCPK1 cells to explore how a second combination of parameters influenced the output of the simulation. We asked whether this second parameter set could generate a more dynamic MT array since it includes a 2-fold greater catastrophe frequency and a 2-fold lower rescue frequency compared to those in Set A (Table 2, Set B; [27]. Nucleation per site was kept at the same rate as that used above. As shown in Fig 3A at 35 μM total tubulin, parameter set B changed the simulation output, although several trends were shared between the two data sets. Parameter set B predicted an array with more, but still infrequent, short MTs. A single MT explored greater space over time, but still only rarely depolymerized completely (Fig 3A, Set B). This parameter set yielded fewer MTs (411 ± 6.4), of slightly shorter average length (20.3 ± 0.1 μm for Set B compared to 22.7 ± 0.1 μm for Set A; p<0.0001) and higher free tubulin concentration (12.5 ± 0.25 μM) compared to values from parameter set A (Fig 3B). The lower number of MTs generated by Set B means that open nucleation sites are present, allowing new MT formation. Applying parameter Set B to the 2-state model and setting nucleation to 0 in the second state demonstrated that MTs were lost over time, an indication that the parameter set allowed occasional complete MT depolymerization. The length of the remaining MTs remained nearly constant, but reached slightly longer lengths as MT number declined (S1 Fig). Approximately half the MTs were lost after about 36,000 s (~ 10 h; S1 Fig). MT numbers were lost in the form of an exponential decay, rather than a linear loss of MTs over time. As discussed below, the slowing of MT loss over time, under the simulation conditions used here, likely resulted because (a) as MTs are lost, the free tubulin concentration rises (to 21 μM at 36,000 s), resulting in faster MT elongation rates; (b) catastrophes, as defined here, occur with a probability based on time, meaning that an individual MT gains more length per unit time when the growth rate is faster; and (c) each MT becomes less likely to completely depolymerize because a longer MT has a greater probability of rescue than does a shorter MT. The rise in free tubulin and the faster growth rate likely account for the slightly longer average MT lengths after new nucleation is blocked. Thus, the model makes the counterintuitive prediction that under some circumstances suppressing nucleation, which by itself would seem to favor net disassembly, can actually lead to a longer and more stable MT array. Thus, the centrosome can potentially serve as a kind of "remote control" system that can modify plus end MT dynamics through indirect mass action-mediated effects. Indeed, these changes to MT dynamics have been observed experimentally in cells harboring mutations in gamma tubulin or other proteins functioning in MT nucleation [28]. Our simulation results are also consistent with conclusions from a previous model [20].

We next asked whether the MT arrays assembled from Set A or Set B would depolymerize when the total tubulin concentration was diluted 10-fold in the second state. Simulations with either parameter set yielded rapid MT depolymerization (S1 Fig). The number of MTs and average MT lengths quickly decreased, with half-times on the order of 100–200 s (about 1.5–3 mins). These values match well with the rate of MT polymer loss from monocytes treated with nocodazole to block assembly [29]. The simulated MT array is surprisingly sensitive to tubulin concentration without the need to invoke a tubulin concentration dependence to catastrophe or rescue frequencies. In the simulated dilution, MTs elongate more slowly and add less polymer per growth phase before undergoing a catastrophe. Although the catastrophe frequency is defined here as a probability per unit time, catastrophes become more likely per μm of polymer as the growth rate slows, raising the effective catastrophe frequency for the growth phase. The slower growth and shorter growth cycles likely account for loss of MT polymer under these simulation conditions. Importantly, these results demonstrate that the simulated array responds to a perturbation that affects plus end dynamics, even with the highly stable array built from parameter Set A. In contrast, setting nucleation rate to zero in State 2 had lesser effects on the array because the plus end dynamics limited complete MT depolymerization at 35 μM total tubulin.

Overall, simulations using parameter set A or B demonstrated that dynamic instability parameters measured at the cell periphery predict an array of MTs that is generally long, with a length distribution shaped as a rising exponential, but also that variation between measured parameter sets yields differences in the shape of the array (probability of short MTs), MT number, average MT length and free tubulin concentration. The two parameter sets responded to tubulin dilution with rapid MT depolymerization, but the simulated arrays did not predict the MT length distribution measured experimentally (Fig 1). To explore how individual parameters contribute to the shape of the MT array, we next applied the Monte Carlo model to simulate MT arrays under varying conditions.

### Lowering total tubulin concentration to 20–30 μM results in more dynamic plus ends

As a first step to examine the roles of individual model parameters in shaping the MT array, we began by looking at how the concentration of total tubulin affects the entire MT array, using either parameter set A or B. These simulations were run with total tubulin concentrations of 20–35 μM. This concentration range was selected because measurements of total tubulin in some systems, such as Xenopus or urchin egg extracts have estimated a total tubulin pool of 20–30 μM [30–32] and because cellular factors could sequester a fraction of total tubulin, reducing the concentration available for assembly below the 35 μM estimate used here. Sequestering of tubulins could occur either through tubulin-binding proteins such as Oncoprotein 18/stathmin [33], or through the generation of non-dynamic, stable MTs [6,34], which essentially act as a tubulin sink, reducing the amount of tubulin available for polymerization.

As shown in Fig 3, lowering the total tubulin from 35 μM to 20–30 μM increased the number of short MTs, and for any single MT, increased the area where the plus end is found over time. For Set A, MTs were no longer confined to a region near the cell boundary and at 20–30 μM tubulin yielded a length distribution resembling that for Set B at 35 μM tubulin. Simulations using Set B yielded the same trend, with more short MTs and fewer total MTs at lower total tubulin concentrations (Fig 3). The average length of MTs, assembled with either parameter set, decreased at lower tubulin concentrations (Fig 3). Overall, these effects likely occur because lowering the free tubulin concentration reduces growth rate in the model. As discussed above, simulated MTs grow more slowly at lower free tubulin concentrations and therefore experience catastrophes more often per unit length polymerized, leading to less net assembly. If the same simple rules are active in cells, sequestering a fraction of the tubulin pool and reducing the concentration available for assembly, will result in fewer MTs, with plus ends localized to wider areas of the cytoplasm. Thus, the model makes the non-intuitive prediction that, under some conditions, a MT destabilizer could indirectly promote dynamic instability and the shape of an entire MT array. Gregoretti et al. [20] reached similar conclusions at lower tubulin concentrations and with a computational model centered on tubulin concentration, rather than on the measured parameters of dynamic instability used here.

### Simulating arrays with a nucleation rate dependent on tubulin concentration

Nucleation rate from centrosomes is predicted to depend on tubulin concentration [24]. Therefore, we tested whether a concentration-dependent nucleation rate would alter MT numbers or lengths. Here we defined nucleation rate as a function of tubulin concentration based on the linear relationship between nucleation rate (EB1-GFP comet emergence from the centrosome) and free tubulin concentration (by incubation in nocodazole as outlined in [24]). Adding this function to the model allowed nucleation rate to vary with available tubulin. Under these conditions, the simulations yielded the near maximum number of MTs (500) using either Set A or Set B and length distributions were similar to those shown in Figs 2 and 3 (35 μM tubulin; data not shown). We realized that these outputs occur because initially, at high free tubulin concentrations, nucleation becomes highly probable and nearly every site nucleates a MT. The array is then rapidly dictated solely by plus end dynamics, which, as shown above, favors stability and infrequent complete shortening of any MT. This means that nucleation quickly becomes a non-factor in the simulations because sites for new MT assembly are not available. We used a linear relationship between nucleation rate and free tubulin concentration as predicted for a free tubulin concentration up to ~ 8–10 μM [24], but it is not yet known experimentally whether the rate continues to rise linearly at higher free tubulin concentrations. Lowering total tubulin to 25 μM and including a concentration-dependent nucleation rate yielded similar results (data not shown).

### Plus end dynamics: Rescue frequency plays the largest role

**Fig 4:**
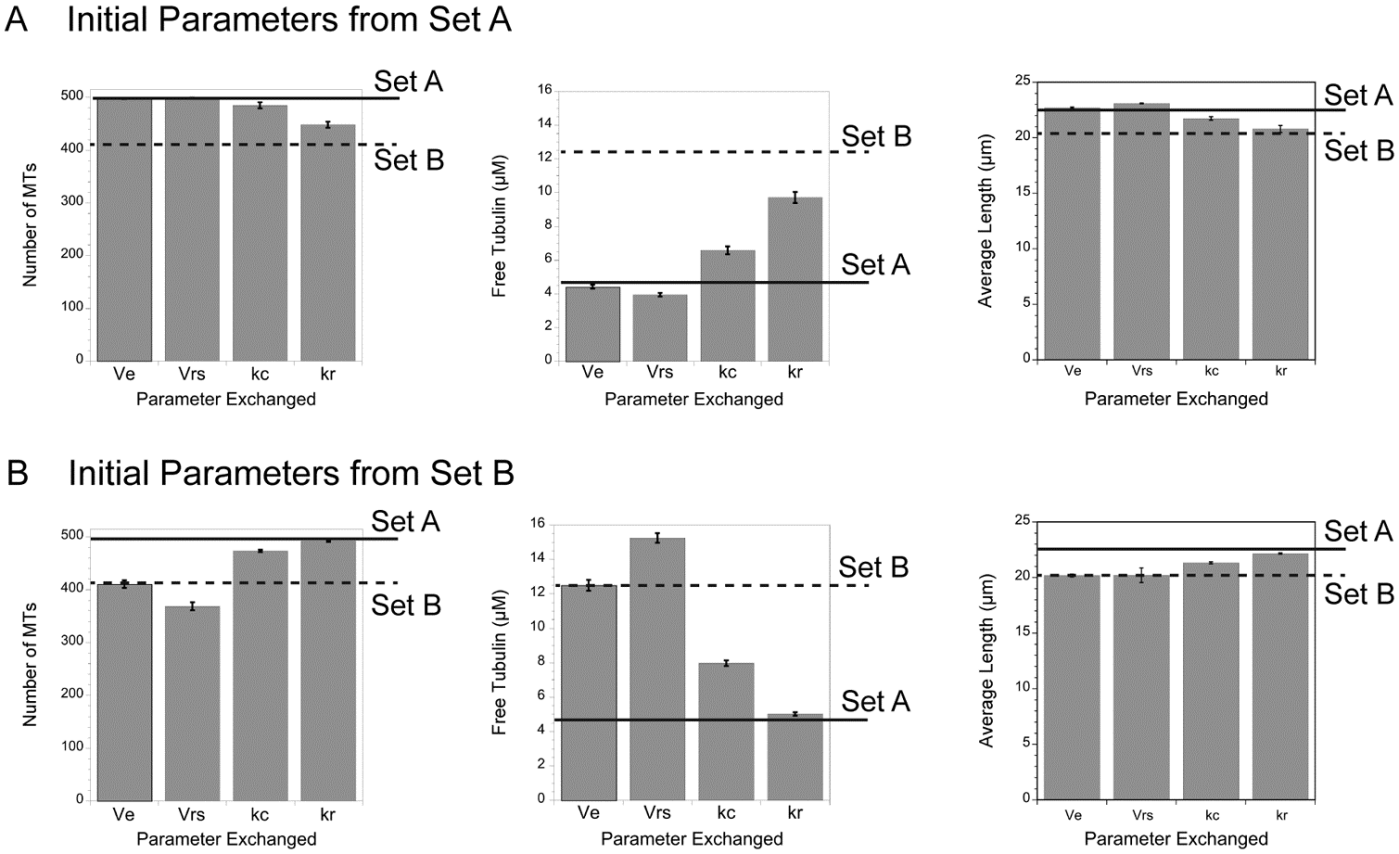
Rescue frequency variations have the largest impact on MT number, length and free tubulin concentration. Simulations from parameter Set A (A) or Set B (B) were run after switching one parameter at a time to the value from the other set. Solid lines show results from the complete Set A, dashed lines from Set B. In (A), switching Set A’s rescue frequency to Set B’s value shifted MT number, average length and free tubulin concentration closest to values predicted by all the parameters in Set B. (B) Starting with Set B and switching to Set A’s rescue frequency resulted in values nearly equal to those predicted by all parameters in Set A. These parameter switches predict that rescue frequency has the largest impact on the shape of the MT array.

Comparing the values for dynamic instability between parameter sets A and B showed that rates of elongation and shortening vary to a small degree, while frequencies of catastrophe and rescue each vary by about 2-fold. To examine how the differences in average values between the parameter sets contributes to the overall MT array, we ran simulations with parameters from one set and systematically switched one parameter at a time to that from the other set. Fig 4 summarizes the results for the predicted numbers of MTs, the free tubulin concentrations and the average MT length. Starting from parameter Set A, switching to the ~ 2-fold lower rescue frequency of set B had the largest impact on the number of MTs, free tubulin and average MT lengths (Fig 4A). The ~ 2-fold higher catastrophe frequency of set B made a smaller impact on MT number, free tubulin and average MT length. Reversing the parameter sets and starting with Set B demonstrated that the lower catastrophe or higher rescue frequencies of Set A generated higher numbers of MTs, reduced the free tubulin and increased average MT length (Fig 4B). Importantly, changing rescue frequency alone was sufficient to switch each output generated by Set B to those generated by Set A. The rate of shortening also varied slightly between the two parameter sets (Table 1; 0.218 μm/sec for Set A vs 0.188 μm/sec for Set B). Switching these rates between the parameter sets also had a small impact on MT number, free tubulin and average MT lengths (Fig 4). The shortening rate likely impacts MT number or free tubulin because we used rescue frequency measured as a function of time, not of length. The faster the MT shortens, the more subunits it will lose per unit time before a rescue occurs. Although shortening rate may add to loss of subunits under the simulation conditions used here, overall the simulations predict that rescue frequency has the largest influence over the shape of the MT array, at least for a range of parameters measured experimentally in epithelial cells.

### Simulating regional differences or variations in dynamic instability

**Fig 5:**
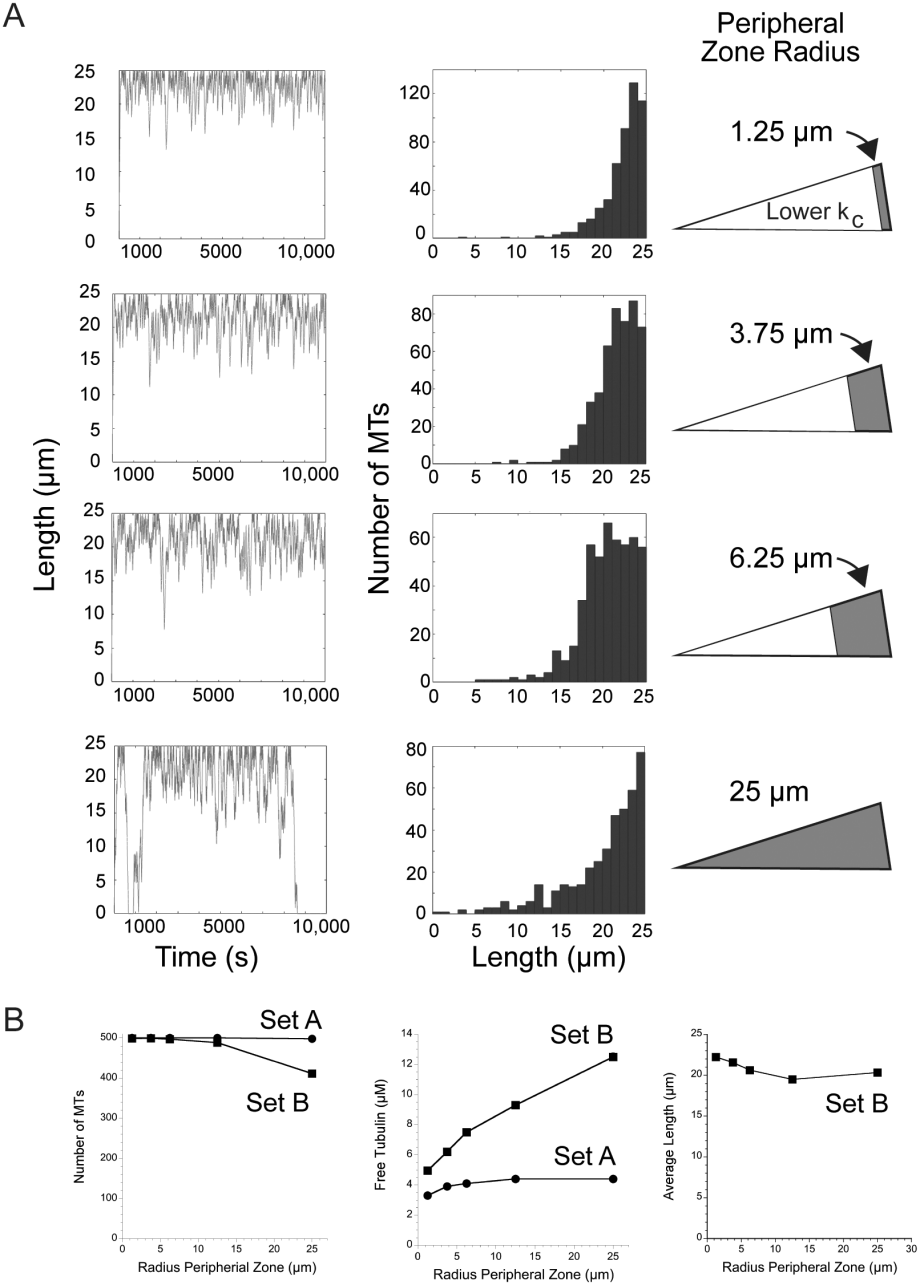
Simulating two cytosolic zones differing in catastrophe frequency. Several groups have documented persistent MT growth (low catastrophe) in the cell interior. We simulated the outcomes of varying widths of an internal stable zone (16x lower catastrophe). (A) Simulations were run using parameter Set B. Data are plotted relative to the width of the peripheral, more dynamic (higher catastrophe) zone (shown in grey in the diagrams). The width of the internal, stable zone excludes MT plus ends because they quickly polymerize through this zone and confines dynamic instability to the more dynamic peripheral zone where catastrophes are much more likely. (B) As the peripheral, dynamic zone becomes narrower (lower values on X axis), Set B parameters predict output values similar to that generated by Set A.

The above simulations considered the cytoplasm as homogenous, without any regional variation in dynamic instability. In contrast, Komarova et al. [10] measured dynamic instability within central and peripheral regions of CHO cells and concluded that the central region of the cell has a greater MT stabilizing environment, with about a 16-fold lower catastrophe frequency in the cell interior, leading to persistent MT growth in this region. The more stable MTs in the cell interior could reflect regional differences in dynamic instability within a cell, or could reflect an age-dependent catastrophe frequency, where catastrophes are rare early in a MT lifetime and become more likely as the MT grows longer [35–38]. Independent of the underlying mechanism, we simulated the effect of a central stabilizing environment relative to a more dynamic zone at the cell periphery by adding to the Monte Carlo model two radii for the central and peripheral zones that together equal the radius of the cell. The catastrophe and/or rescue frequencies can be defined within each zone. Komarova et al. [10] did not detect a difference in rescue frequency between different regions of the cell and therefore we kept rescue constant between zones and examined only the consequences of a central MT stabilizing zone of lower catastrophe frequency. For these simulations we varied the radius of the peripheral region from 1.25 μm to 25 μm, where the latter value recapitulates the simulations above, with dynamic instability unchanged by position in the cell. For comparison, Komarova et al. [10] estimated that the radius of the peripheral, more dynamic zone equals ~15% of total cell radius, or 3.75 μm for our simulated cell of 25 μm radius. As shown in Fig 5A for parameter set B, the width of the more dynamic, peripheral zone determines the width of the area where MT ends are found, or essentially the area that MT plus ends will explore. Larger radii for the central stabilizing area resulted in fewer short MTs and concentrated MT ends to a narrower region at the cell periphery. Under these conditions, MTs are highly unlikely to ever depolymerize completely. For parameter Set B, the number of MTs approximated the maximum of 500 as the more stabilizing central environment extended to about half of the cell radius (Fig 5B). By promoting a greater number of MTs, a greater central stabilizing environment (smaller peripheral zone) also reduced the amount of free tubulin because more tubulin is tied up in existing polymer and unavailable for assembly (Fig 5B). The average length of MTs also increased when the peripheral, more dynamic zone was at the smallest radius (Fig 5B). In contrast, for parameter Set A, running simulations to vary the width of the peripheral and central zones had little effect on MT number or free tubulin because the parameters favor growth to the cell periphery even in the absence of a more stabilizing central environment (Fig 5B). For this parameter set, MTs grow persistently in the cell interior and extend to the boundary, where contact with the cell edge contributes significantly to the frequency of catastrophe, and the high frequency of rescue returns shortening MTs back into a growth phase (see Fig 2).

The above simulations used averages for all measured parameters, yet variability in rates or frequencies is typically described for dynamic instability of MTs assembled from purified tubulins or within cells. Most strikingly, frequencies of catastrophe and rescue often have standard deviations equal to the mean, or nearly so [8,9,39]. Likewise, for a living cell, the position of the cell margin is not fixed in space but can change position, even in a stationary cell. Therefore, the algorithm was modified so that shortening rate, catastrophe and rescue frequencies and/or boundary position can shift randomly, within a defined range, to a new value at each step in the simulation, which effectively introduced noise into the model parameters. We used parameter Set B for these simulations because they allow room for the system to generate either more or less stable MT arrays. Shortening rate was varied in a range covering one standard deviation. For catastrophe and rescue, which often have reported large standard deviations relative to the mean, we varied these frequencies in a range between 0 and a maximum equal to twice the mean value; this variation encompasses the largest symmetrical variation around the mean value, without having the lowest value fall below zero.

**Fig 6:**
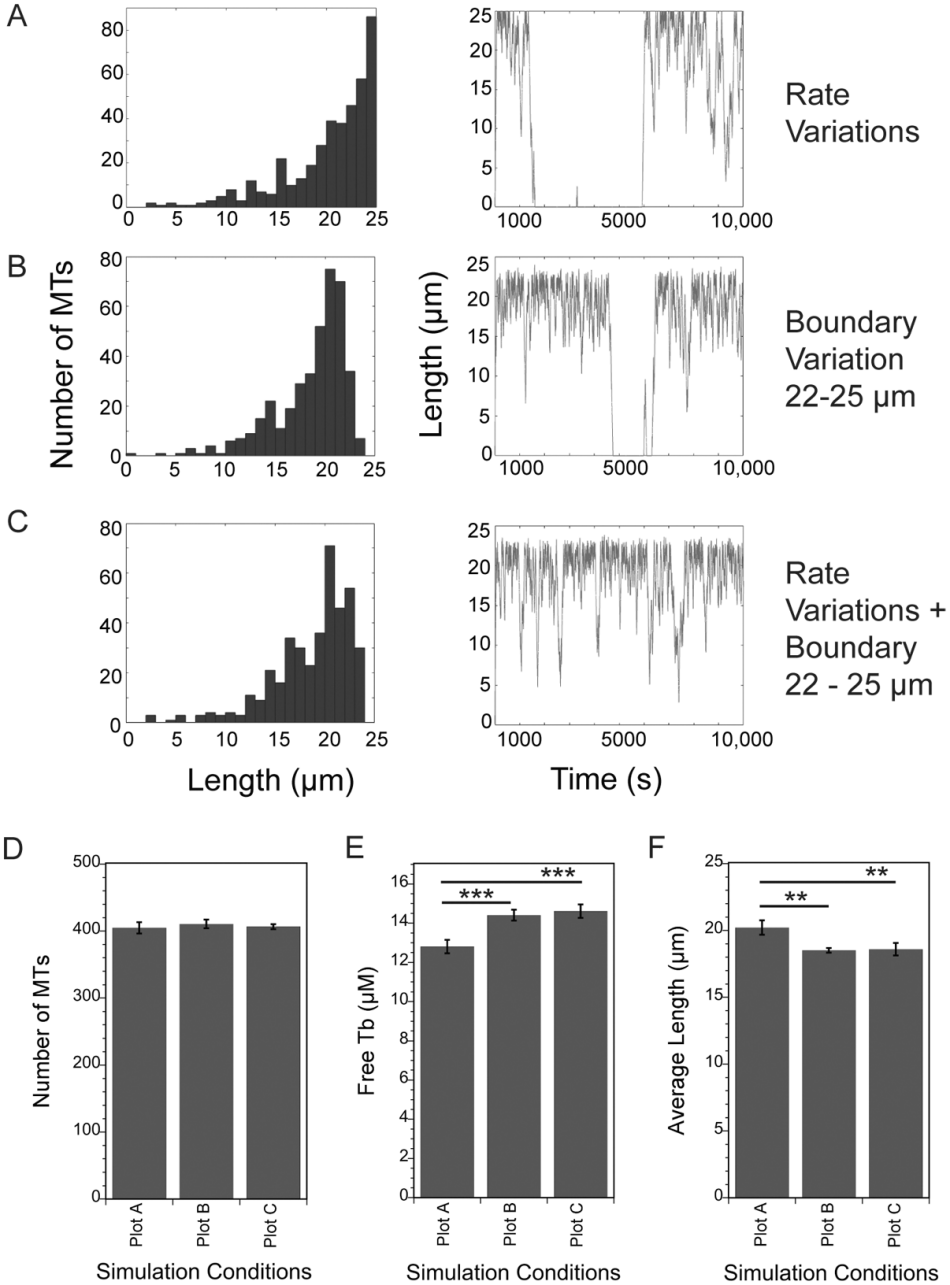
Varying the position of the cell boundary shifts the length distribution, raises free tubulin concentration and shortens average MT length without changing MT number. The algorithm was modified to allow some parameters to vary randomly during a simulation. (A) Dynamic instability parameters were varied randomly by ± 1 sd around the means as described in the text. (B) The cell radius was reduced randomly by 0–3 μm to simulate small shifts in position of the cell boundary. (C) Combination of conditions in (A,B). See text for details. (D-F) The number of MTs is constant under the conditions shown in A-C, but free tubulin is slightly greater, and average length slightly shorter when the cell radius is reduced randomly by 0–3 μm during simulations. *** p< 0.0001, ** p = 0.001.

Adding variation in cell boundary position and/or in shortening, catastrophe and rescue rates demonstrated that a shifting position of the cell boundary changes the distribution of MT lengths, while variations in dynamic instability parameters do not. Allowing shortening, catastrophe and rescue to vary about their averages did not change the MT length distribution (compare distribution in Fig 6A to that in Fig 3 for Set B at 35 μM tubulin) and had minimal impact on MT number or free tubulin concentration (not shown). In contrast, shifting the position of the boundary by 1–3 μm resulted in MTs that less frequently reached all the way to the maximum length of 25 μm and instead reached slightly shorter maximum lengths (Fig 6B shows a 3 μm variation in cell radius). Varying position of the cell margin also resulted in length distributions that rise gradually to about 20 μm and then fall off sharply near the cell margin. Allowing the position of the boundary to shift over a 3 μm range did not change the number of MTs, but free tubulin increased and average MT lengths decreased (Fig 6D–6F). The position of the boundary was the dominant factor in these simulations since combining a shifting boundary with variations in the dynamic instability of each MT yielded similar results to those observed after only changing the position of the boundary. Note that for MTs within a cell, a changing radius could represent either extension/contraction of the plasma membrane and/or shifts in the position of the centrosome near the center of the cell. Either of these events could contribute to the shape of the MT length distribution.

Length distributions generated by allowing the position of the cell margin to change position were similar, but not identical, to the distribution in LLCPK1 interphase cells (Fig 1). These data indicate that the position of the cell boundary, and its ability to stimulate catastrophe are critical to defining the shape of the MT array (see also [20,40]). We confirmed that the position of the cell boundary was the major determinant in the lengths of MTs by simulating a much larger cell, with a radius of 250 μm, and found that MTs still grew to the cell periphery with the same shaped distribution, only at much longer lengths (parameter Set A; not shown). Our coarse-grained simulation is independent of mechanism, and uses only measured catastrophe frequencies, which should include catastrophes stimulated by MT ends hitting the cell margin. That we need to include boundary-stimulated catastrophes in the simulations suggests that the catastrophe frequencies measured in cells are underestimates of the actual rate, possibly due to cycles of short sequences of growth, catastrophe and rescue as MTs reach the cell membrane. A high rate of rescue at the cell margin was also suggested recently by Seetapun et al. [23], based on the length of the GTP-tubulin cap at MT ends as measured in LLCPK1 cells. Here, a high rescue frequency is possible within the tail of a long plus end GTP-tubulin cap [23]. In this scenario, MTs reaching the cell membrane will bounce rapidly between catastrophe induced by the cell boundary, and rescue within the back end of the GTP-tubulin cap, until the cap is fully lost and more extensive depolymerization ensues.

### Simulation of MT disassembly during the cell cycle transition from interphase to prophase

To apply the model to a cell cycle change to the MT array, we simulated the transition from interphase to prophase (around the time of nuclear envelope breakdown (NEBD)), using published values [9]. We used Set A for the interphase parameters to set up the array in state 1 and then switched to prophase parameters (Table 1) in state 2. We chose to use Set A interphase parameters because these values were published together with those for prophase [9]. For the first set of simulations we kept the nucleation rate constant at its interphase rate throughout states 1 and 2. We did not run the prophase state for long durations because this state is short lived in cells and within mins switches to mitotic rates and frequencies [9]. Before describing the simulation results, it is useful to consider what experimental observations predict. Previous measurements of MT polymer as a percentage of the total tubulin pool showed that MT polymer decreases from ~70% of total tubulin in interphase to ~20% of total tubulin concurrent with NEBD [39,40]. For a cell with 35 μM total tubulin and assembled into an interphase array, we expect that simulating a switch to prophase dynamics should yield ~ 28 μM free tubulin in a short amount of time. Based on unpublished observations, we estimate that LLCPK1 cells disassemble the interphase array in 5 mins (300 s) or less.

**Fig 7:**
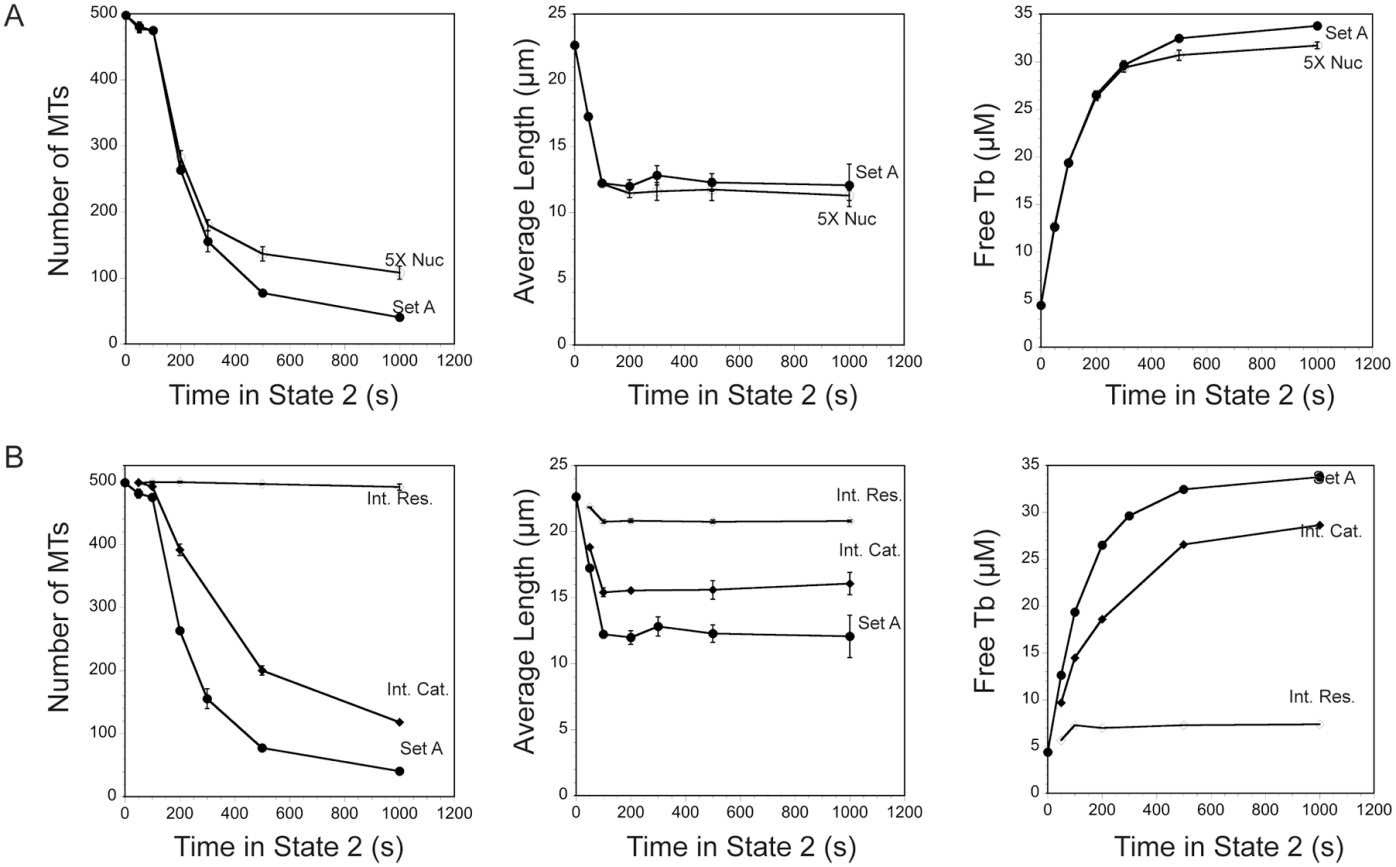
Simulating MT array dissolution from interphase to prophase (NEBD). (A) Parameter Set A was used to build an interphase array and then dynamic instability was switched to parameters measured at around NEBD (Table 2). The parameters measured at NEBD promote rapid loss of MTs at rates consistent with the rapid process estimated LLCPK1 cells. Increasing nucleation rate 5-fold, as measured at around NEBD in these cells [22] did not make a significant contribution to the shape of the array. (B) Simulating the switch to prophase dynamics, but keeping either catastrophe or rescue at their interphase rates showed that keeping rescue at its interphase rate was sufficient to maintain the interphase array and prevent array dissolution, indicating that the reduced rescue at prophase is the critical parameter allowing disassembly of the MT array as cells prepare to enter mitosis.

As shown in Fig 7A, switching an interphase array to values measured in prophase causes a rapid loss of the MT array. The number of MTs drops quickly with a half time of about 200 s (3.3 mins). Average length drops rapidly to ~ 12 μm in 100 s and stays at that value up to 1,000 s in state 2. Likewise, free tubulin rises rapidly, and reaches ~28 μM after ~ 250–300 s (5 mins), indicating that the simulations match well with experimental observations [41,42].

The simulations above kept nucleation at a single probability per site for states 1 and 2, but nucleation rate also increases about 5-fold around the time of NEBD [22] and the increased nucleation rate could offset some of the MT polymer loss described above. In cells, centrosome maturation includes increases in size of the centrosome, amount of gamma tubulin localized to the centrosome, and centrosomal nucleation rate [43]. The increased rate of nucleation in prophase and mitosis is thought to be due to an increase in the number of nucleation sites, but our present model does not allow us to increase the number of nucleation sites in the second state. Instead, we mimicked the 5-fold increase in nucleation per centrosome by increasing the probability of nucleation 5-fold at each site in state 2. Simulations again predicted a rapid loss of MT numbers and average length, concomitant with a rapid rise in free tubulin concentration (Fig 7A). These simulations demonstrate that changes to plus end dynamic instability at around the time of NEBD are sufficient to depolymerize the interphase array within mins, even with a 5-fold increase in nucleation rate. While the simulations are blind to molecular mechanisms underlying the changes to plus end dynamics, they indicate that additional mechanisms, such as MT severing along MT lengths or at their ends [44,45] or MT release from the centrosome and minus end depolymerization are not necessary for rapid clearance of the interphase MT array in preparation for mitosis.

Finally, we used the simulations to predict which change to MT plus end dynamics at NEBD, increased catastrophe frequency or decreased rescue frequency, plays a larger role in clearing the interphase MT array. Measured frequencies showed an ~ 3-fold increase in catastrophe and an ~7.5-fold decrease in rescue at NEBD, compared to the frequencies measured in interphase. Rates of growth and shortening were nearly identical between the two cell cycle times (Table 2). We used the 2-state simulations to build the interphase array in state 1, then switched to parameters measured near NEBD in state 2, but kept either catastrophe or rescue at their interphase value. As shown in Fig 7B, keeping rescue frequency at the interphase value is sufficient to prevent dissolution of the interphase array. MT number, average MT length and free tubulin concentration show little change from their interphase values after 1,000 s (~17 mins). Keeping catastrophe at its interphase frequency slowed dissolution of the interphase array, for example approximately doubling the half-time for MT loss, but still permitted extensive loss of polymer, with free tubulin reaching ~ 28 μM at 1,000 s. Taken together, these simulations predict that decreased rescue frequency is critical for dissolution of the interphase array in preparation for mitosis. Previously, Gliksman et al. [19] reached a similar conclusion, demonstrating that decreased rescue frequency is the critical parameter change needed to build a mitotic array of short MTs compared to the long MTs of interphase. Our simulations predict that mitotic entry requires either a cell cycle dependent activation of a rescue inhibitor, or inactivation of rescue-promoting proteins, to allow rapid dissolution of the interphase array. To our knowledge, the only specific rescue inhibitor is EMAP, a protein originally isolated from echinoderms [46]. Interestingly, this protein is phosphorylated by CDK1 [47], possibly reflecting a cell-cycle dependent activation of rescue-inhibiting activity, which would allow rapid loss of MTs upon mitotic entry. A mechanism to explain EMAP’s rescue-inhibiting activity is unknown and at this point it is only speculation that its activity rises at NEBD. A more likely explanation for lower rescue frequency at NEBD is reduced activity of a rescue promoter, lowering the probability of rescue at mitotic entry. Rescue promoters include CLIP-170 and CLASP [2,48,49], but whether these proteins are transiently turned off at NEBD is not yet known. Interestingly, expression of a truncated CLIP-170 to block endogenous CLIP-170 activity in interphase cells decreased rescue frequency by 7-fold [48], nearly identical to the 7.5-fold decrease measured in prophase cells [7]. Komarova et al. [48] found that decreasing rescue to this degree decreased MT lifetime from ~20 min to ~ 1 min, which also agrees with our simulations demonstrating the critical role of decreased rescue in depolymerizing much of the interphase array (Fig 7B).

#### Conclusions

Here we described the results from a relatively simple Monte Carlo model that allows one to explore the consequences of individual parameters of plus end dynamic instability, tubulin concentration, and nucleation rate on the lengths and numbers of MTs per cell as well as the free tubulin concentration. The model can be run to simulate a single state (one parameter set) or can build an array in one state and then examine the consequences of a change in parameter(s) in a second state. The simulations yielded several unexpected results. First, changes in the total tubulin concentration dictate the total number of MTs and the free tubulin concentration, with little impact on average MT length, indicating that any mechanism that sequesters tubulins or stores them in stable MTs will impact the remaining MT array (Fig 3). Second, rescue frequency is the critical parameter regulating MT instability at mitotic entry (Fig 7B) or within interphase cells (Fig 4). Additionally, we found that the position of the cell boundary made a significant contribution to MT catastrophes, beyond the frequency measured experimentally, consistent with previous models of dynamic instability in a confined space [20,40], and that shifts in the position of the boundary could account for the lower number of MT ends at the extreme edge of the cell, as shown experimentally (Fig 1; [50]). Finally, our simulations, as well as those others [3,20], demonstrate that the shape of the MT array and the number of MTs within the cell can shift tubulin partioning between monomer and polymer pools. That shifts in plus end MT dynamics or the rate of MT nucleation can raise or lower the free tubulin concentration supports an idea originally proposed by Mitchison and Kirschner [3] postulating that an equilibrium critical concentration of tubulin subunits is unlikely to exist in cells; instead tubulin partitioning between monomer and polymer pools varies with nucleation and MT dynamics. As suggested originally by Mitchison and Kirschner [3], a variable tubulin subunit concentration could be sensed by cells, providing feedback on the status of the MT cytoskeleton and/or possibly relaying downstream signals.

In summary, the model we describe here provides a good mimic for cell-based processes such as mitotic entry (explored here) or cell locomotion at leading and trailing edges (e.g. [15]) using commonly measured MT dynamics parameters. We see the model as useful both for predicting the shape of MT arrays under various conditions or changing conditions from commonly measured experimental parameters, and additionally as a teaching tool to allow students to explore each parameter of dynamic instability within a larger context.

## Methods

### Cell culture, fixation and immunofluorescence

LLCPK1 cells or a stable LLCPK1 cell line stably expressing EB1-GFP were maintained as previously described [25; the parent LLCPK1 cell line was obtained from the ATCC and the EB1-GFP sub-line was generated from the parental line]. For the data shown in Fig 1, cells were trypsinized and cultured on coverslips coated to generate defined adhesive patterns (Cytoo, Bethesda, MD). Cells were allowed to attach to coverslips for 4 h before fixation in -20°C methanol/EDTA [25]. Coverslips were labeled with primary antibodies: mouse anti-EB1 (BD Transduction Laboratories) and rabbit anti-gamma-tubulin (AK-15; Sigma-Aldrich) followed by secondary antibodies (Goat anti-mouse IgG-Alexa 488; Goat anti-rabbit-Alex 568; Molecular Probes/Invitrogen). Images used here were collected from single cells attached to large disc (22.6 μm radius) adhesive spots. Images were collected using a Zeiss 880 confocal microscope, 63X plan-apo objective, at a Z depth of 0.5 μm. The optical section closest to the coverslip surface was used for the length measurements. MT lengths were measured as straight lines between the center of the centrosome (gamma tubulin focus) and the end of the EB1 at the MT tip.

### Monte Carlo simulations

Code was written in MATLAB and is given in S1 Appendix. A general description of the simulation conditions and parameters is given in Results and Discussion. The model is coarse-grained at the molecular level, because it is computationally expensive and unnecessarily detailed to include molecular resolution. Briefly noted here are the rules and assumptions that are incorporated into the simulations: (1) MTs are represented as straight elements composed of a linear chain of subunits representing the length of the MT without incorporating molecular detail of tubulin dimer, protofilament or MT structure. We retained the correct dimension that 1 μm of MT length is made up of 1624 dimers; (2) MT growth rate is dependent on the soluble tubulin dimer concentration, where V_g_ = k_on_ [Tub]+C1; we used an estimated k_on_ = 1/60 μm/s per μM and a y-intercept (C1) at zero based on data from [8,21], but these values can be changed within the simulation. Note that the k_on_ defined here is a phenomenological k_on_ for the entire MT, not the microscopic k_on_ for the single protofilaments estimated by Gardner et al. [36]; (3) Tubulin-GDP, released by depolymerization, is immediately converted to tubulin-GTP and competent to polymerize without delay.

For all results shown here, simulation of any single condition was run 4–10 times. For each simulation we recorded values for MT number, average MT length and free tubulin (μM). MATLAB-generated plots of MT length distributions and an example life history plot for a single MT were also recorded. Results for each parameter set were highly reproducible with small standard deviations. Plots were generated in Kaleidagraph, which was also used for statistical analyses (t-test).

## Supporting information

S1 Fig Simulations shifting an interphase array to to a second state with either no new nucleation or total tubulin diluted 10-fold. Initial arrays were built from Set A or Set B at 35 μM total tubulin. (A) After 10,000 s in state 1, nucleation rate was set to 0. Loss of MTs over time in the second state reflects complete MT depolymerization. New MTs cannot replace the lost MTs because nucleation has been eliminated. MTs dynamics from Set A yield MTs that do not depolymerize appreciably after 48 hrs in state 2. In contrast, MT dynamics from Set B yields a loss of MTs over time. For the remaining MTs, the average MT length increases slightly, likely due to a higher free tubulin concentration as some MTs depolymerize. (B) The two state model was also used to simulate dilution of total tubulin to 3.5 μM. MTs assembled from parameter Set A or B rapidly depolymerized over several mins, as measured by the number of MTs or the average MT length.
(TIF)

S1 Appendix S1 Appendix contains the algorithm used here, coded in MATLAB. Parameters are defined in the text or in the notes included in the appendix.
(DOCX)
