# Supplementary figures and images for: Monte Carlo simulations of microtubule arrays: The critical roles of rescue transitions, the cell boundary, and tubulin concentration in shaping microtubule distributions
Source: PLoS One. 2018 May 21;13(5):e0197538. doi: 10.1371/journal.pone.0197538 (PMC5962052; doi:10.1371/journal.pone.0197538)

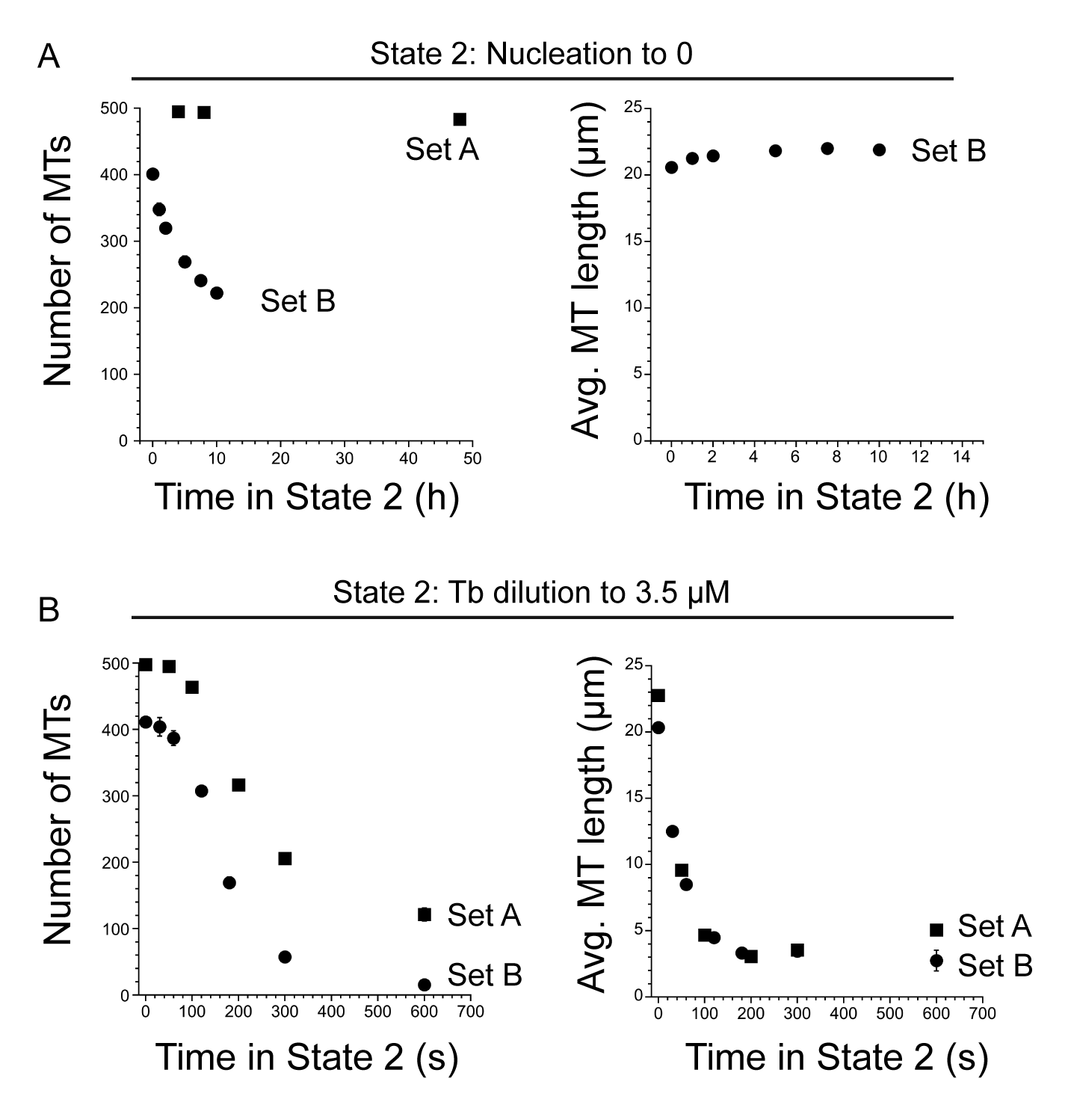

Supplement: S1 Fig — Initial arrays were built from Set A or Set B at 35 μM total tubulin. (A) After 10,000 s in state 1, nucleation rate was set to 0. Loss of MTs over time in the second state reflects complete MT depolymerization. New MTs cannot replace the lost MTs because nucleation has been eliminated. MTs dynamics from Set A yield MTs that do not depolymerize appreciably after 48 hrs in state 2. In contrast, MT dynamics from Set B yields a loss of MTs over time. For the remaining MTs, the average MT length increases slightly, likely due to a higher free tubulin concentration as some MTs depolymerize. (B) The two state model was also used to simulate dilution of total tubulin to 3.5 μM. MTs assembled from parameter Set A or B rapidly depolymerized over several mins, as measured by the number of MTs or the average MT length. (TIF) [file pone.0197538.s001.tif]
