# Supplementary material for: Monte Carlo simulations of microtubule arrays: The critical roles of rescue transitions, the cell boundary, and tubulin concentration in shaping microtubule distributions
Source: PLoS One. 2018 May 21;13(5):e0197538. doi: 10.1371/journal.pone.0197538 (PMC5962052; doi:10.1371/journal.pone.0197538)
Supplement: S1 Appendix — Parameters are defined in the text or in the notes included in the appendix. (DOCX) [file pone.0197538.s002.docx]

S1 Appendix. MATLAB code used to simulate microtubule arrays in a cell-like environment.

clear

% define parameters

Nmtmax=500; % number of MTs max

Vcell=1000; % volume of the cell (µm3)

TubulinTotalConc=35; % total concentration of tubulin (µM)

TubulinTotalNumb=TubulinTotalConc*(602)*Vcell; % total # of tubulins

knuc=0.0005; % nucleation rate (s-1)

Vg=0.192; % growth rate (µm/s)

% Make Vg dependent on tubulin concentration, Vg=kon*[tub]+C1

kon=1/60; % slope (µm/s per µM)

C1=0; % intercept (µm/s)

% mean shortening rate (µm/s)

    % meanVs=0.218; % shortening rate (µm/s)

% Vs extremes

Vsmin=0.218;

Vsmax=0.218;

% mean catastrophe & mean rescue

    % kcint=0.026; % interior catastrophe freq (s-1)

    % kcper=0.026; % periphery catastrophe freq (s-1)

    % kresint=0.175; % interior rescue freq (s-1)

    % kresper=0.175; % periphery rescue freq (s-1)

% kcat & kres extremes

kcintmin=0.026;

kcintmax=0.026;

kcpermin=0.026;

kcpermax=0.026;

kresintmin=0.175;

kresintmax=0.175;

krespermin=0.175;

krespermax=0.175;

% cell radius extremes

Rcellmin=25; % cell radius minimum (µm)

Rcellmax=25; % cell radius maximum (µm)

% peripheral zone extremes

Rpermin=10; % periphery length minimum (µm)

Rpermax=10; % periphery length maximum (µm)

dt=1; % time step (s)

Nsteps=1000; % number of steps

Lg=Vg*dt; % length added in one step (µm)

pnuc=1-exp(-knuc*dt); % probability of nucleation

Lmt=zeros(1,Nmtmax); % initialize MT lengths

state=ones(1,Nmtmax); % initialize MT states

for j=1:Nsteps

    % update shortening rate (µm/s)

    % select rand values b/w extremes

    Vs=Vsmin+(Vsmax-Vsmin).*rand(1,1);

    Ls=Vs*dt; % length lost in one step (µm)

    % update kcat & kres

    % select rand values b/w extremes

    kcint=kcintmin+(kcintmax-kcintmin).*rand(1,1);

    kcper=kcpermin+(kcpermax-kcpermin).*rand(1,1);

    kresint=kresintmin+(kresintmax-kresintmin).*rand(1,1);

    kresper=krespermin+(krespermax-krespermin).*rand(1,1);

    pcint=1-exp(-kcint*dt); % probability of catastrophe in cell interior

    pcper=1-exp(-kcper*dt); % probability of catastrophe in cell periphery

    presint=1-exp(-kresint*dt); % probability of catastrophe in cell interior

    presper=1-exp(-kresper*dt); % probability of catastrophe in cell periphery

    % update cell radius

    % select rand values b/w extremes

    Rcell=Rcellmin+(Rcellmax-Rcellmin).*rand(1,1);

    % update zones

    % Rper select rand value b/w extremes

    Rper=Rpermin+(Rpermax-Rpermin).*rand(1,1); % periphery length (µm)

    Rint=Rcell-Rper; % cell interior length (µm)

    for i=1:Nmtmax

        if state(i)==0 %test for nucleation

            if rand<pnuc

                state(i)=1; %put into growing state if nucleated

                Lmt(i)=Lg;

            end

        elseif state(i)==1 %for growing mts

            Lmt(i)=Lmt(i)+Lg;

            if Lmt(i)<Rint

                if rand<pcint

                    state(i)=2;

                end

            elseif Lmt(i)>Rint

                if rand<pcper

                    state(i)=2;

                end

            end

        elseif state(i)==2

            Lmt(i)=Lmt(i)-Ls;

            if Lmt(i)<Rint

                if rand<presint

                    state(i)=1;

                end

            elseif Lmt(i)>Rint

                if rand<presper

                    state(i)=1;

                end

            end

        end

        if Lmt(i)>Rcell %check boundaries

            Lmt(i)=Rcell;

            state(i)=2;

        elseif Lmt(i)<0

            Lmt(i)=0;

            state(i)=0;

        end

        LLmt(i,j)=Lmt(i);

        sstate(i,j)=state(i);

        Lmtmean(j)=mean(LLmt(:,j));

        Lmtstd(j)=std(LLmt(:,j));

        if i==1

            Lmtone(j)=Lmt(i);

            ttime(j)=dt*j;

        end

    end

    % update free tubulin concentration

    TotalMTlength=sum(Lmt); %µm

    TubulinFreeNumb=TubulinTotalNumb-1624*TotalMTlength;

    Tub(j)=TubulinFreeNumb/(602*Vcell); % free tubulin conc (µM)

    Vg=kon*Tub(j)+C1;

    Lg=Vg*dt;

end

% RE-define parameters

Nmtmax=500; % number of MTs max

Vcell=1000; % volume of the cell (µm3)

TubulinTotalConc=35; % total concentration of tubulin (µM)

TubulinTotalNumb=TubulinTotalConc*(602)*Vcell; % total # of tubulins

knuc=0.0005; % nucleation rate (s-1)

Vg=0.192; % growth rate (µm/s)

% Make Vg dependent on tubulin concentration, Vg=kon*[tub]+C1

kon=1/60; % slope (µm/s per µM)

C1=0; % intercept (µm/s)

% mean shortening rate (µm/s)

    % meanVs=0.218; % shortening rate (µm/s)

% Vs extremes

Vsmin=0.218;

Vsmax=0.218;

% mean catastrophe & mean rescue

    % kcint=0.075; % interior catastrophe freq (s-1)

    % kcper=0.075; % periphery catastrophe freq (s-1)

    % kresint=0.023; % interior rescue freq (s-1)

    % kresper=0.023; % periphery rescue freq (s-1)

% kcat & kres extremes

kcintmin=0.026;

kcintmax=0.026;

kcpermin=0.026;

kcpermax=0.026;

kresintmin=0.105;

kresintmax=0.105;

krespermin=0.105;

krespermax=0.105;

% cell radius extremes

Rcellmin=25; % cell radius minimum (µm)

Rcellmax=25; % cell radius maximum (µm)

% peripheral zone extremes

Rpermin=10; % periphery length minimum (µm)

Rpermax=10; % periphery length maximum (µm)

dt=1; % time step (s)

Nsteps2=1000; % number of steps

Lg=Vg*dt; % length added in one step (µm)

pnuc=1-exp(-knuc*dt); % probability of nucleation

% Lmt=zeros(1,Nmtmax); % initialize MT lengths

% Use last set of Lmt from the upper loop

% state=ones(1,Nmtmax); % initialize MT states

for j=Nsteps+1:Nsteps+Nsteps2 % pick up where we left off above

    % update shortening rate (µm/s)

    % select rand values b/w extremes

    Vs=Vsmin+(Vsmax-Vsmin).*rand(1,1);

    Ls=Vs*dt; % length lost in one step (µm)

    % update kcat & kres

    % select rand values b/w extremes

    kcint=kcintmin+(kcintmax-kcintmin).*rand(1,1);

    kcper=kcpermin+(kcpermax-kcpermin).*rand(1,1);

    kresint=kresintmin+(kresintmax-kresintmin).*rand(1,1);

    kresper=krespermin+(krespermax-krespermin).*rand(1,1);

    pcint=1-exp(-kcint*dt); % probability of catastrophe in cell interior

    pcper=1-exp(-kcper*dt); % probability of catastrophe in cell periphery

    presint=1-exp(-kresint*dt); % probability of catastrophe in cell interior

    presper=1-exp(-kresper*dt); % probability of catastrophe in cell periphery

    % update cell radius

    % select rand values b/w extremes

    Rcell=Rcellmin+(Rcellmax-Rcellmin).*rand(1,1);

    % update zones

    % Rper select rand value b/w extremes

    Rper=Rpermin+(Rpermax-Rpermin).*rand(1,1); % periphery length (µm)

    Rint=Rcell-Rper; % cell interior length (µm)

    for i=1:Nmtmax

        if state(i)==0 %test for nucleation

            if rand<pnuc

                state(i)=1; %put into growing state if nucleated

                Lmt(i)=Lg;

            end

        elseif state(i)==1 %for growing mts

            Lmt(i)=Lmt(i)+Lg;

            if Lmt(i)<Rint

                if rand<pcint

                    state(i)=2;

                end

            elseif Lmt(i)>Rint

                if rand<pcper

                    state(i)=2;

                end

            end

        elseif state(i)==2

            Lmt(i)=Lmt(i)-Ls;

            if Lmt(i)<Rint

                if rand<presint

                    state(i)=1;

                end

            elseif Lmt(i)>Rint

                if rand<presper

                    state(i)=1;

                end

            end

        end

        if Lmt(i)>Rcell %check boundaries

            Lmt(i)=Rcell;

            state(i)=2;

        elseif Lmt(i)<0

            Lmt(i)=0;

            state(i)=0;

        end

        LLmt(i,j)=Lmt(i);

        sstate(i,j)=state(i);

        Lmtmean(j)=mean(LLmt(:,j));

        Lmtstd(j)=std(LLmt(:,j));

        if i==1

            Lmtone(j)=Lmt(i);

            ttime(j)=dt*j;

        end

    end

    % update free tubulin concentration

    TotalMTlength=sum(Lmt); %µm

    TubulinFreeNumb=TubulinTotalNumb-1624*TotalMTlength;

    Tub(j)=TubulinFreeNumb/(602*Vcell); % free tubulin conc (µM)

    Vg=kon*Tub(j)+C1;

    Lg=Vg*dt;

end

figure(1)

plot(ttime,Lmtone)

count=0;

for i=1:Nmtmax

    if state(i)==0

    else

        count=count+1;

        length(count)=Lmt(i);

    end

end

count

lengthmean=mean(length)

figure(2)

xbins=0.5:24.5; %extreme centers for equally spaced bins

nbins=25; % number of bins

hist(length,xbins,nbins) %plot histogram of lengths with above

xlim([0 25])

xlabel('length (µm)')

ylabel('frequency (#MTs)')

title ('MT length distribution')

figure(3)

plot(ttime,Lmtmean)

figure(4)

plot(ttime,Tub)

ac=length';
